# Supplementary material for: Machine Learning Accelerates Crystallization for Structure Determination
Source: Angew Chem Int Ed Engl. 2026 May 4;65(25):e1218503. doi: 10.1002/anie.1218503 (PMC13266958; doi:10.1002/anie.1218503)

checkCIF/PLATON report

Structure factors have been supplied for datablock(s) lcz060

THIS REPORT IS FOR GUIDANCE ONLY. IF USED AS PART OF A REVIEW PROCEDURE FOR PUBLICATION, IT SHOULD NOT REPLACE THE EXPERTISE OF AN EXPERIENCED CRYSTALLOGRAPHIC REFEREE.

No syntax errors found. CIF dictionary Interpreting this report

Datablock: lcz060

Bond precision:	C-C = 0.0190 A		Wavelength=1.54184
Cell:	a=12.4289(2)	b=13.0611(2)	c=23.2463(3)
	alpha=86.696(1)	beta=88.837(1)	gamma=76.537(1)
Temperature:	100 K		

	Calculated	Reported
Volume	3663.80(10)	3663.80(10)
Space group	P -1	P -1
Hall group	-P 1	-P 1
Moiety formula	2(C15 H3 Ag3 F18 N6), 2(C13 H8 O3), 3(C H4 O)	2(C15 H3 Ag3 F18 N6), 2(C13 H8 O3), 3(C H4 O)
Sum formula	C59 H34 Ag6 F36 N12 O9	C59 H34 Ag6 F36 N12 O9
Mr	2386.20	2386.20
Dx,g cm-3	2.163	2.163
Z	2	2
Mu (mm-1)	14.044	14.044
F000	2300.0	2300.0
F000’	2310.98	
h,k,lmax	15,16,29	15,16,29
Nref	15638	14861
Tmin,Tmax	0.182,0.185	0.494,1.000
Tmin’	0.081	

Correction method= # Reported T Limits: Tmin=0.494 Tmax=1.000
AbsCorr = MULTI-SCAN

Data completeness= 0.950 Theta(max)= 77.833

R(reflections)= 0.0877(11810) wR2(reflections)= 0.2023(14861)

S = 1.043 Npar= 1107

The following ALERTS were generated. Each ALERT has the format
test-name_ALERT_alert-type_alert-level.
Click on the hyperlinks for more details of the test.

Alert level B

```
PLAT971_ALERT_2_B Check Calcd Resid. Dens.  0.99Ang From N01C           3.07 eA-3
```

Author Response: This Alert is due to presence of residual density in the presence of heavy metal atom (Ag).

```
PLAT971_ALERT_2_B Check Calcd Resid. Dens.  0.99Ang From N00Y           2.58 eA-3
```

Author Response: This Alert is due to presence of residual density in the presence of heavy metal atom (Ag).

```
PLAT971_ALERT_2_B Check Calcd Resid. Dens.  1.02Ang From N00M           2.51 eA-3
```

Author Response: This Alert is due to presence of residual density in the presence of heavy metal atom (Ag).

Alert level C

```
PLAT094_ALERT_2_C Ratio of Maximum / Minimum Residual Density ....       2.05 Report
PLAT213_ALERT_2_C Atom F02W           has ADP max/min Ratio .....        3.2 prolat
PLAT234_ALERT_4_C Large Hirshfeld Difference F02F     --C02Y     .       0.17 Ang.
PLAT234_ALERT_4_C Large Hirshfeld Difference N00M     --C02C     .       0.19 Ang.
PLAT342_ALERT_3_C Low Bond Precision on  C-C Bonds ..............     0.01898 Ang.
PLAT790_ALERT_4_C Centre of Gravity not Within Unit-Cell: Resd.  #          1 Note
              C15 H3 Ag3 F18 N6
PLAT906_ALERT_3_C Large K Value in the Analysis of Variance ......     17.106 Check
PLAT906_ALERT_3_C Large K Value in the Analysis of Variance ......      4.033 Check
PLAT906_ALERT_3_C Large K Value in the Analysis of Variance ......      2.038 Check
PLAT911_ALERT_3_C Missing FCF Refl Between Thmin & STh/L=    0.600         65 Report
               13  1  0,  13  2  0,   1  4  0, -14 -3  1,  -1  0  1,  13  1  1,
               -5 13  1, -14 -3  2, -12 -3  2,  -8  5  2,  -7 10  2, -12 -3  3,
               14  0  3,   6-12  5, -14 -2  5,  11 -4  8,  -8 10  8,  -7 11  8,
              -12  2  9,  14  3  9,  -9  9  9,  -8  9  9,  -8 10  9,  -7 10  9,
               -7 11  9, -12  2 10,  -9  8 10,  -8  9 10,  -7  9 10,  -7 10 10,
                   (   35 More Missing: see the .ckf listing file)
PLAT971_ALERT_2_C Check Calcd Resid. Dens.  0.84Ang From Ag05           2.19 eA-3
```

Author Response: This Alert is due to presence of residual density in the presence of heavy metal atom (Ag).

```
PLAT971_ALERT_2_C Check Calcd Resid. Dens.  0.92Ang From Ag02           1.89 eA-3
```

Author Response: This Alert is due to presence of residual density in the presence of heavy metal atom (Ag).

```
PLAT971_ALERT_2_C Check Calcd Resid. Dens.  0.95Ang From Ag03           1.82 eA-3
```

Author Response: This Alert is due to presence of residual density in the presence of heavy metal atom (Ag).

```
PLAT971_ALERT_2_C Check Calcd Resid. Dens.  0.89Ang From Ag01           1.72 eA-3
```

Author Response: This Alert is due to presence of residual density in the presence of heavy metal atom (Ag).

```
PLAT971_ALERT_2_C Check Calcd Resid. Dens.  0.88Ang From Ag01           1.71 eA-3
```

Author Response: This Alert is due to presence of residual density in the presence of heavy metal atom (Ag).

```
PLAT971_ALERT_2_C Check Calcd Resid. Dens.  1.08Ang From Ag04           1.71 eA-3
```

Author Response: This Alert is due to presence of residual density in the presence of heavy metal atom (Ag).

```
PLAT971_ALERT_2_C Check Calcd Resid. Dens.  1.00Ang From F020           1.61 eA-3
```

Author Response: This Alert is due to presence of residual density in the presence of heavy metal atom (Ag).

```
PLAT971_ALERT_2_C Check Calcd Resid. Dens.  0.77Ang From Ag02           1.60 eA-3
```

Author Response: This Alert is due to presence of residual density in the presence of heavy metal atom (Ag).

```
PLAT971_ALERT_2_C Check Calcd Resid. Dens.  1.10Ang From Ag05           1.58 eA-3
```

Author Response: This Alert is due to presence of residual density in the presence of heavy metal atom (Ag).

```
PLAT971_ALERT_2_C Check Calcd Resid. Dens.  1.11Ang From Ag06           1.58 eA-3
```

Author Response: This Alert is due to presence of residual density in the presence of heavy metal atom (Ag).

```
PLAT971_ALERT_2_C Check Calcd Resid. Dens.  1.08Ang From N00P           1.58 eA-3
```

Author Response: This Alert is due to presence of residual density in the presence of heavy metal atom (Ag).

```
PLAT971_ALERT_2_C Check Calcd Resid. Dens.  1.08Ang From Ag01           1.53 eA-3
```

Author Response: This Alert is due to presence of residual density in the presence of heavy metal atom (Ag).

```
PLAT972_ALERT_2_C Check Calcd Resid. Dens.  0.82Ang From Ag05          -1.72 eA-3
PLAT972_ALERT_2_C Check Calcd Resid. Dens.  0.65Ang From Ag06          -1.54 eA-3
PLAT972_ALERT_2_C Check Calcd Resid. Dens.  0.58Ang From Ag05          -1.53 eA-3
PLAT977_ALERT_2_C Check Negative Difference Density on H019      .     -0.53 eA-3
PLAT977_ALERT_2_C Check Negative Difference Density on H01Z      .     -0.44 eA-3
PLAT977_ALERT_2_C Check Negative Difference Density on H02P      .     -0.40 eA-3
```

Alert level G

```
PLAT003_ALERT_2_G Number of Uiso or U(i,j)  Restrained non-H-Atoms        11 Report
PLAT007_ALERT_5_G Number of Unrefined Donor-H Atoms .............          5 Report
              H01I  H01E  H01G  H01Q  H01W
PLAT083_ALERT_2_G SHELXL Second Parameter in WGHT  Unusually Large     145.88 Why ?
PLAT154_ALERT_1_G The s.u.'s on the Cell Angles are Equal ..(Note)      0.001 Degree
PLAT186_ALERT_4_G The CIF-Embedded .res File Contains ISOR Records         3 Report
PLAT242_ALERT_2_G Low    'MainMol' Ueq as Compared to Neighbors of       C022 Check
PLAT242_ALERT_2_G Low    'MainMol' Ueq as Compared to Neighbors of       C029 Check
PLAT242_ALERT_2_G Low    'MainMol' Ueq as Compared to Neighbors of       C02K Check
PLAT242_ALERT_2_G Low    'MainMol' Ueq as Compared to Neighbors of       C02V Check
PLAT242_ALERT_2_G Low    'MainMol' Ueq as Compared to Neighbors of       C031 Check
PLAT242_ALERT_2_G Low    'MainMol' Ueq as Compared to Neighbors of       C03E Check
PLAT242_ALERT_2_G Low    'MainMol' Ueq as Compared to Neighbors of       C02O Check
PLAT242_ALERT_2_G Low    'MainMol' Ueq as Compared to Neighbors of       C02Y Check
PLAT242_ALERT_2_G Low    'MainMol' Ueq as Compared to Neighbors of       C036 Check
PLAT242_ALERT_2_G Low    'MainMol' Ueq as Compared to Neighbors of       C038 Check
PLAT242_ALERT_2_G Low    'MainMol' Ueq as Compared to Neighbors of       C03A Check
PLAT242_ALERT_2_G Low    'MainMol' Ueq as Compared to Neighbors of       C03D Check
PLAT434_ALERT_2_G Short Inter HL..HL Contact F00L     ..F00W     .       2.79 Ang.
                                               -1-x,1-y,1-z  =      2_466 Check
PLAT720_ALERT_4_G Number of Unusual/Non-Standard Labels ..........        155 Note
              Ag01    Ag02    Ag03    Ag04    Ag05    Ag06    F007    F008
              F009    F00A    F00B    O00C    F00D    O00E    N00F    F00G
              F00H    F00I    F00J    N00K    F00L    N00M    F00N    N00O
              N00P    O00Q    N00R    F00S    N00T    N00U    F00V    F00W
              F00X    N00Y    F00Z    N010    F011    O012    F013    F014
              N015    C016    F017    C018    C019    H019    F01A    F01B
              N01C    O01E    H01E    C01F    H01F    O01G    H01G    F01H
```

```
              O01I     H01I     C01J     H01J     C01K     C01L     F01M     C01N
              F01O     F01P     O01Q     H01Q     C01R     F01S     C01T     C01U
              C01V     H01V     O01W     H01W     C01X     H01X     C01Y     H01Y
              C01Z     H01Z     F020     C021     H021     C022     F023     F024
              C025     C026     C027     C028     C029     C02A     H02A     C02B
              C02C     F02D     C02E     H02E     F02F     C02G     H02G     C02H
              C02I     H02I     F02J     C02K     C02L     C02M     C02N     C02O
              C02P     H02P     C02Q     C02R     H02R     C02S     H02S     C02T
              H02T     C02U     C02V     F02W     C02X     C02Y     C02Z     H02Z
              C030     C031     C032     H032     C033     H033     C034     H034
              C035     H03G     H03H     H03I     C036     C037     C038     F039
              C03A     C03B     H03D     H03E     H03F     C03C     H03A     H03B
              H03C     C03D     C03E
PLAT790_ALERT_4_G Centre of Gravity not Within Unit-Cell: Resd.  #          2 Note
              C15 H3 Ag3 F18 N6
PLAT790_ALERT_4_G Centre of Gravity not Within Unit-Cell: Resd.  #          3 Note
              C13 H8 O3
PLAT790_ALERT_4_G Centre of Gravity not Within Unit-Cell: Resd.  #          4 Note
              C13 H8 O3
PLAT790_ALERT_4_G Centre of Gravity not Within Unit-Cell: Resd.  #          5 Note
              C H4 O
PLAT790_ALERT_4_G Centre of Gravity not Within Unit-Cell: Resd.  #          6 Note
              C H4 O
PLAT790_ALERT_4_G Centre of Gravity not Within Unit-Cell: Resd.  #          7 Note
              C H4 O
PLAT860_ALERT_3_G Number of Least-Squares Restraints ............         66 Note
PLAT883_ALERT_1_G Absent Datum for _atom_sites_solution_primary ..     Please Do !
PLAT910_ALERT_3_G Missing FCF Reflection(s) Below Theta(Min)[Deg]=       3.48 Note
                0  0  1,
PLAT912_ALERT_4_G Missing # of FCF Reflections Above STh/L=  0.600        709 Note
PLAT913_ALERT_3_G Missing # of Very Strong Reflections in FCF ....          1 Note
                1  4  0,
PLAT933_ALERT_2_G Number of HKL-OMIT Records in Embedded .res File         10 Note
               -4  9 19,  -3  9 20,  -1  9 21,  -5  9 12,  -3  8 23,  -6  9 14,
               -6  9 13, -10  2 16,  -6  8 20,  11 -4  8,
PLAT941_ALERT_3_G Average HKL Measurement Multiplicity ..........         2.4 Low
PLAT969_ALERT_5_G The 'Henn et al.' R-Factor-gap value ..........       3.449 Note
              Predicted wR2: Based on SigI**2  5.86 or SHELX Weight 19.41
PLAT978_ALERT_2_G Number C-C Bonds with Positive Residual Density.          0 Info
```

```
   0 ALERT level A = Most likely a serious problem - resolve or explain
   3 ALERT level B = A potentially serious problem, consider carefully
  28 ALERT level C = Check. Ensure it is not caused by an omission or oversight
  34 ALERT level G = General information/check it is not something unexpected

   2 ALERT type 1 CIF construction/syntax error, inconsistent or missing data
  40 ALERT type 2 Indicator that the structure model may be wrong or deficient
   9 ALERT type 3 Indicator that the structure quality may be low
  12 ALERT type 4 Improvement, methodology, query or suggestion
   2 ALERT type 5 Informative message, check
```

Validation response form

Please find below a validation response form (VRF) that can be filled in and pasted into your CIF.

```
# start Validation Reply Form
_vrf_PLAT094_lcz060
;
PROBLEM: Ratio of Maximum / Minimum Residual Density ....       2.05 Report
RESPONSE: ...
;
_vrf_PLAT213_lcz060
;
PROBLEM: Atom F02W            has ADP max/min Ratio .....        3.2 prolat
RESPONSE: ...
;
_vrf_PLAT234_lcz060
;
PROBLEM: Large Hirshfeld Difference F02F     --C02Y     .       0.17 Ang.
RESPONSE: ...
;
_vrf_PLAT342_lcz060
;
PROBLEM: Low Bond Precision on  C-C Bonds ..............    0.01898 Ang.
RESPONSE: ...
;
_vrf_PLAT790_lcz060
;
PROBLEM: Centre of Gravity not Within Unit-Cell: Resd.  #          1 Note
RESPONSE: ...
;
_vrf_PLAT906_lcz060
;
PROBLEM: Large K Value in the Analysis of Variance ......     17.106 Check
RESPONSE: ...
;
_vrf_PLAT911_lcz060
;
PROBLEM: Missing FCF Refl Between Thmin & STh/L=    0.600         65 Report
RESPONSE: ...
;
_vrf_PLAT972_lcz060
;
PROBLEM: Check Calcd Resid. Dens.  0.82Ang From Ag05           -1.72 eA-3
RESPONSE: ...
;
_vrf_PLAT977_lcz060
;
PROBLEM: Check Negative Difference Density on H019      .      -0.53 eA-3
RESPONSE: ...
;
# end Validation Reply Form
```

It is advisable to attempt to resolve as many as possible of the alerts in all categories. Often the minor alerts point to easily fixed oversights, errors and omissions in your CIF or refinement strategy, so attention to these fine details can be worthwhile. It is up to the individual to critically assess their own results and, if necessary, seek expert advice.

PLATON version of 17/09/2025; check.def file version of 17/09/2025

duplicate check

No duplication found

Datablock lcz060 - ellipsoid plot

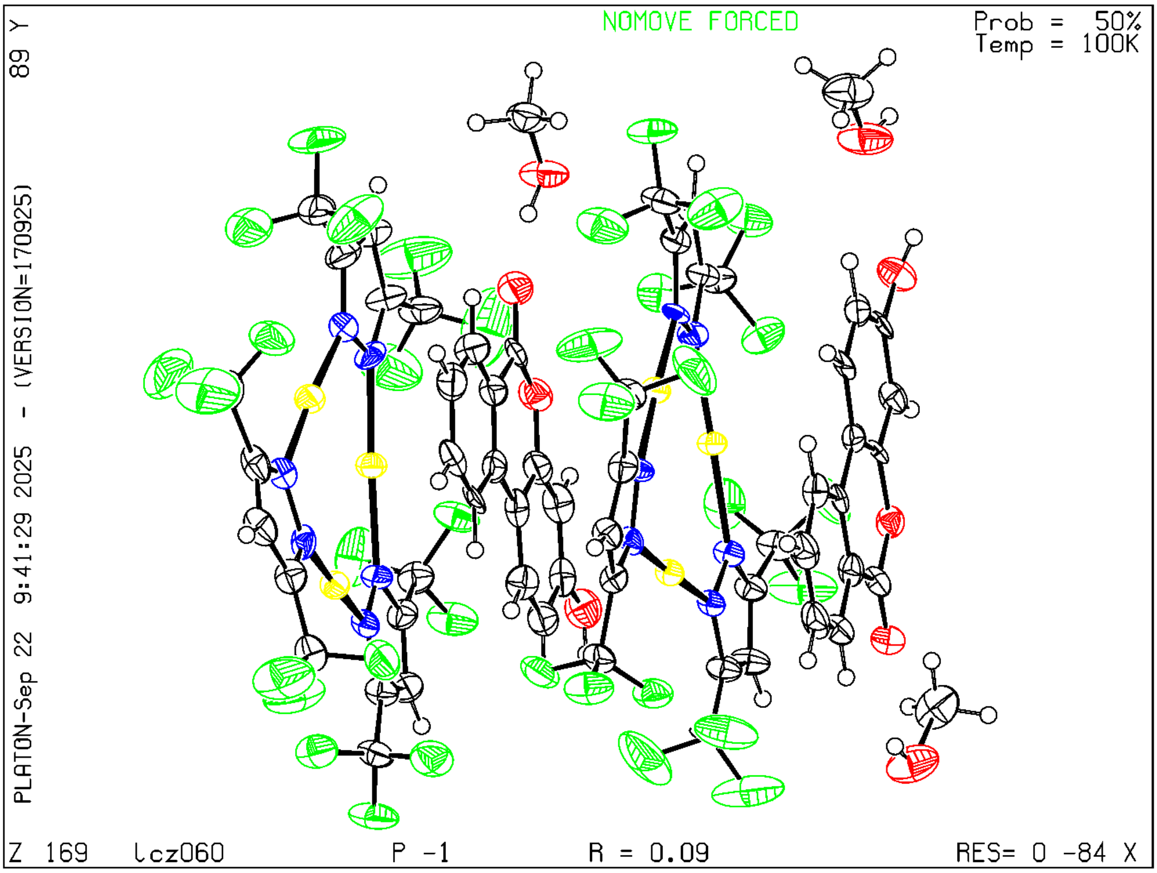

Supplement: Supplementary file 2 — Supporting File 2: anie72462‐sup‐0002‐DataFile.zip. [file ANIE-65-e1218503-s002.zip › supplementary_materials_crystallographic_data submit.zip!/cif_and_checkcif(1-114)/Ag3Pz3_60/Ag3Pz3_60_checkcif.pdf]
